# [5,10,15,20-Tetra­kis(4-tol­yl)porphyrin]zinc(II) dichloro­methane solvate

**DOI:** 10.1107/S1600536810019963

**Published:** 2010-06-05

**Authors:** Sean McGill, Vladimir N. Nesterov, Stephanie L. Gould

**Affiliations:** a Department of Chemistry, Austin College, 900 North Grand, Sherman, TX 75090-4400, USA; b Department of Chemistry, University of North Texas, Denton, TX 76203-5017, USA

## Abstract

In the title complex, [Zn(C_48_H_36_N_4_)]·CH_2_Cl_2_, the Zn^II^ atom lies on an inversion center and the dichloro­methane solvent mol­ecule is disordered around an inversion center. The tolyl substituents are twisted compared to the central aromatic ring system of the porphyrin, similar to what is seen in previously published structures of this molecule [Dastidar & Goldberg (1996 ▶). *Acta Cryst.* C**52**, 1976–1980]. The dihedral angles between the mean planes of the tolyl rings and the central ring are 66.98 (6) and 60.40 (6)°.

## Related literature

For other solvates of this mol­ecule see: Dastidar & Goldberg (1996 ▶). For similar structures of ligand-bridged porphyrin sandwich-type structures, see: Diskin-Posner *et al.* (2002 ▶); Mak *et al.* (1998 ▶); Kieran *et al.* (2005 ▶); Dastidar *et al.* (1996 ▶). For the synthesis of the title compound, see: Adler *et al.* (1967 ▶). 
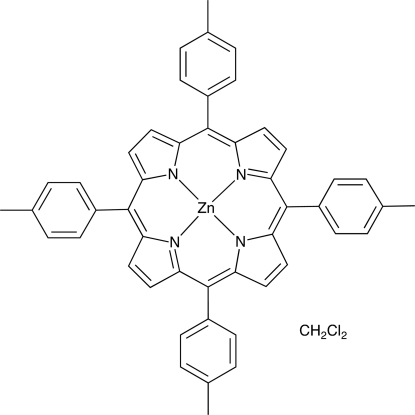


## Experimental

### 

#### Crystal data

[Zn(C_48_H_36_N_4_)]·CH_2_Cl_2_
*M* *_r_* = 819.10
Monoclinic, 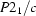

*a* = 14.349 (2) Å
*b* = 8.5273 (14) Å
*c* = 15.637 (3) Å
β = 94.995 (2)°
*V* = 1906.1 (5) Å^3^
*Z* = 2
Mo *K*α radiation
μ = 0.83 mm^−1^
*T* = 100 K
0.16 × 0.13 × 0.09 mm

#### Data collection

Bruker SMART APEXII CCD diffractometer
Absorption correction: numerical (*SADABS*; Bruker, 2001 ▶) *T*_min_ = 0.880, *T*_max_ = 0.930
16249 measured reflections
3901 independent reflections
3533 reflections with *I* > 2σ(*I*)
*R*_int_ = 0.026

#### Refinement

*R*[*F* ^2^ > 2σ(*F* ^2^)] = 0.042
*wR*(*F* ^2^) = 0.094
*S* = 1.00
3901 reflections
261 parameters
1 restraint
H-atom parameters constrained
Δρ_max_ = 0.95 e Å^−3^
Δρ_min_ = −1.23 e Å^−3^

### 

Data collection: *APEX2* (Bruker, 2007 ▶); cell refinement: *SAINT* (Bruker, 2007 ▶); data reduction: *SAINT*; program(s) used to solve structure: *SHELXTL* (Sheldrick, 2008 ▶); program(s) used to refine structure: *SHELXTL*; molecular graphics: *SHELXTL*; software used to prepare material for publication: *SHELXTL*.

## Supplementary Material

Crystal structure: contains datablocks global, I. DOI: 10.1107/S1600536810019963/nk2033sup1.cif

Structure factors: contains datablocks I. DOI: 10.1107/S1600536810019963/nk2033Isup2.hkl

Additional supplementary materials:  crystallographic information; 3D view; checkCIF report

## Figures and Tables

**Fig. 1.**
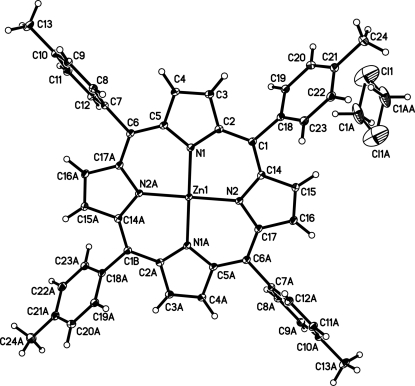
Ellipsoid plot of zinc 5,10,15,20-tetrakis(4'-tolyl)porphyrin (50% probability displacement ellipsoids). Unlabled atoms are related to labeled atoms by inversion symmetry.

## supplementary crystallographic information

### Comment

While pursing ligand bridged porphyrin sandwich-type supramolecular structures, similar to those created by Diskin-Posner *et al.* (2002), Mak *et al.* (1998) and Kieran *et al.* (2005), we sought to crystallize zinc 5,10,15,20-tetrakis(4'-tolyl)porphyrin with pyrazine. The resulting deep red crystals that formed were found not to contain any pyrazine, but contained a well ordered porphyrin structure different than those previously published (Dastidar & Goldberg, 1996). The role of the pyrazine in the crystal deposition is unknown and will be explored further as the same crystals can not be obtained without the compound being present.

The title porphyrin (Figure 1) has a zinc atom located at the center of the porphyrin framework in near exact planarity to the porphyrin. The tolyl substituents are angled compared to the central aromatic ring of the porphyrin, similar to what is seen in previously published structures (Dastidar & Goldberg, 1996). The dihedral angles between the mean planes of the peripheral rings to the central ring are 66.98 (6) and 60.40 (6) °.

### Experimental

The synthesis of zinc 5,10,15,20-tetrakis(4'-tolyl)porphyrin was carried out according literature procedures (Adler *et al.*, 1967). Dark red crystals were grown by liquid diffusion of methanol into a dichloromethane solution containing 20 mg of zinc 5,10,15,20-tetrakis(4'- tolyl)porphyrin and 2 mg of pyrazine.

### Refinement

The dichloromethane solvent molecule is disordered around a center of inversion. Non-hydrogen atoms were refined anisotropically with an occupation factor of 0.5 for C and 1.0 for Cl. All C-bound H atoms were placed in idealized positions (C—H = 0.95–1.00 Å) and allowed to ride on their parent atoms. H atoms were constrained so that *U*_iso_(H) were equal to 1.2Ueq or 1.5Ueq of their respective parent atoms.

### Crystal data

**Table:**

[Zn(C_48_H_36_N_4_)]·CH_2_Cl_2_	*F*(000) = 848
*M**_r_* = 819.10	*D*_x_ = 1.427 Mg m^−^^3^
Monoclinic, *P*2_1_/*c*	Mo *K*α radiation, λ = 0.71073 Å
Hall symbol: -P 2ybc	Cell parameters from 8831 reflections
*a* = 14.349 (2) Å	θ = 2.6–26.1°
*b* = 8.5273 (14) Å	µ = 0.83 mm^−^^1^
*c* = 15.637 (3) Å	*T* = 100 K
β = 94.995 (2)°	Block, red
*V* = 1906.1 (5) Å^3^	0.16 × 0.13 × 0.09 mm
*Z* = 2	

### Data collection

**Table:**

Bruker SMART APEXII CCD diffractometer	3901 independent reflections
Radiation source: fine-focus sealed tube	3533 reflections with *I* > 2σ(*I*)
graphite	*R*_int_ = 0.026
ω scans	θ_max_ = 26.4°, θ_min_ = 2.6°
Absorption correction: numerical (*SADABS*; Bruker, 2001)	*h* = −17→17
*T*_min_ = 0.880, *T*_max_ = 0.930	*k* = −10→10
16249 measured reflections	*l* = −19→19

### Refinement

**Table:**

Refinement on *F*^2^	Primary atom site location: structure-invariant direct methods
Least-squares matrix: full	Secondary atom site location: difference Fourier map
*R*[*F*^2^ > 2σ(*F*^2^)] = 0.042	Hydrogen site location: inferred from neighbouring sites
*wR*(*F*^2^) = 0.094	H-atom parameters constrained
*S* = 1.00	*w* = 1/[σ^2^(*F*_o_^2^) + (0.020*P*)^2^ + 5.*P*] where *P* = (*F*_o_^2^ + 2*F*_c_^2^)/3
3901 reflections	(Δ/σ)_max_ = 0.002
261 parameters	Δρ_max_ = 0.95 e Å^−^^3^
1 restraint	Δρ_min_ = −1.23 e Å^−^^3^

### Special details

**Table:**

Geometry. All e.s.d.'s (except the e.s.d. in the dihedral angle between two l.s. planes) are estimated using the full covariance matrix. The cell e.s.d.'s are taken into account individually in the estimation of e.s.d.'s in distances, angles and torsion angles; correlations between e.s.d.'s in cell parameters are only used when they are defined by crystal symmetry. An approximate (isotropic) treatment of cell e.s.d.'s is used for estimating e.s.d.'s involving l.s. planes.
Refinement. Refinement of *F*^2^ against ALL reflections. The weighted *R*-factor *wR* and goodness of fit *S* are based on *F*^2^, conventional *R*-factors *R* are based on *F*, with *F* set to zero for negative *F*^2^. The threshold expression of *F*^2^ > σ(*F*^2^) is used only for calculating *R*-factors(gt) *etc*. and is not relevant to the choice of reflections for refinement. *R*-factors based on *F*^2^ are statistically about twice as large as those based on *F*, and *R*- factors based on ALL data will be even larger.

### Fractional atomic coordinates and isotropic or equivalent isotropic displacement parameters (Å^2^)

**Table:**

	*x*	*y*	*z*	*U*_iso_*/*U*_eq_	Occ. (<1)
Zn1	0.5000	0.5000	0.5000	0.01504 (11)	
N1	0.43558 (13)	0.5556 (2)	0.38246 (11)	0.0164 (4)	
N2	0.37660 (13)	0.5259 (2)	0.55317 (11)	0.0164 (4)	
C1	0.27573 (15)	0.6038 (3)	0.42370 (14)	0.0168 (4)	
C2	0.34261 (16)	0.5909 (3)	0.36365 (14)	0.0170 (4)	
C3	0.32403 (16)	0.6157 (3)	0.27249 (14)	0.0193 (5)	
H3A	0.2649	0.6380	0.2428	0.023*	
C4	0.40637 (16)	0.6012 (3)	0.23723 (14)	0.0187 (5)	
H4A	0.4163	0.6145	0.1784	0.022*	
C5	0.47622 (16)	0.5618 (3)	0.30559 (14)	0.0167 (4)	
C6	0.57093 (15)	0.5358 (3)	0.29533 (14)	0.0163 (4)	
C7	0.60335 (15)	0.5564 (3)	0.20727 (14)	0.0165 (4)	
C8	0.57431 (16)	0.4550 (3)	0.14006 (14)	0.0182 (5)	
H8A	0.5322	0.3721	0.1496	0.022*	
C9	0.60651 (16)	0.4740 (3)	0.05923 (14)	0.0197 (5)	
H9A	0.5861	0.4036	0.0143	0.024*	
C10	0.66818 (16)	0.5946 (3)	0.04310 (14)	0.0208 (5)	
C11	0.69649 (17)	0.6956 (3)	0.11008 (15)	0.0215 (5)	
H11A	0.7385	0.7785	0.1005	0.026*	
C12	0.66440 (16)	0.6775 (3)	0.19122 (14)	0.0194 (5)	
H12A	0.6844	0.7485	0.2359	0.023*	
C13	0.7035 (2)	0.6148 (3)	−0.04444 (15)	0.0301 (6)	
H13A	0.7502	0.6989	−0.0422	0.045*	
H13B	0.6511	0.6420	−0.0863	0.045*	
H13C	0.7321	0.5167	−0.0617	0.045*	
C14	0.29318 (16)	0.5754 (3)	0.51224 (14)	0.0171 (4)	
C15	0.22477 (16)	0.5933 (3)	0.57396 (14)	0.0189 (5)	
H15A	0.1622	0.6288	0.5625	0.023*	
C16	0.26662 (16)	0.5501 (3)	0.65116 (14)	0.0191 (5)	
H16A	0.2384	0.5474	0.7039	0.023*	
C17	0.36176 (15)	0.5088 (3)	0.63887 (14)	0.0167 (4)	
C18	0.17871 (15)	0.6502 (3)	0.39095 (14)	0.0181 (5)	
C19	0.16187 (16)	0.7945 (3)	0.35042 (14)	0.0207 (5)	
H19A	0.2122	0.8654	0.3455	0.025*	
C20	0.07234 (17)	0.8357 (3)	0.31716 (15)	0.0246 (5)	
H20A	0.0626	0.9335	0.2887	0.030*	
C21	−0.00341 (17)	0.7362 (3)	0.32472 (15)	0.0247 (5)	
C22	0.01316 (17)	0.5947 (3)	0.36693 (15)	0.0241 (5)	
H22A	−0.0378	0.5260	0.3739	0.029*	
C23	0.10252 (16)	0.5513 (3)	0.39923 (15)	0.0208 (5)	
H23A	0.1120	0.4531	0.4273	0.025*	
C24	−0.10064 (18)	0.7807 (4)	0.28820 (18)	0.0340 (6)	
H24A	−0.1464	0.7423	0.3262	0.051*	
H24B	−0.1133	0.7336	0.2312	0.051*	
H24C	−0.1053	0.8951	0.2835	0.051*	
Cl1	−0.00291 (18)	1.13537 (18)	0.44337 (11)	0.1434 (9)	
C1A	0.0459 (5)	0.9997 (10)	0.5120 (5)	0.081 (3)	0.50
H1AA	0.0352	1.0697	0.5615	0.098*	0.50
H1AB	0.0987	0.9580	0.4859	0.098*	0.50

### Atomic displacement parameters (Å^2^)

**Table:**

	*U*^11^	*U*^22^	*U*^33^	*U*^12^	*U*^13^	*U*^23^
Zn1	0.01529 (18)	0.01885 (19)	0.01128 (17)	0.00098 (14)	0.00281 (13)	0.00119 (14)
N1	0.0164 (9)	0.0193 (9)	0.0138 (9)	0.0001 (7)	0.0031 (7)	0.0008 (7)
N2	0.0166 (9)	0.0203 (10)	0.0125 (9)	0.0004 (7)	0.0022 (7)	0.0014 (7)
C1	0.0173 (11)	0.0175 (11)	0.0157 (10)	−0.0008 (9)	0.0020 (8)	0.0009 (9)
C2	0.0181 (11)	0.0176 (11)	0.0154 (10)	−0.0011 (9)	0.0014 (8)	0.0007 (8)
C3	0.0199 (11)	0.0226 (12)	0.0150 (11)	0.0003 (9)	−0.0001 (9)	0.0007 (9)
C4	0.0213 (11)	0.0217 (12)	0.0132 (10)	0.0009 (9)	0.0017 (8)	0.0010 (9)
C5	0.0203 (11)	0.0165 (11)	0.0136 (10)	−0.0008 (9)	0.0026 (8)	0.0001 (8)
C6	0.0192 (11)	0.0159 (11)	0.0139 (10)	−0.0013 (8)	0.0033 (8)	−0.0006 (8)
C7	0.0166 (10)	0.0198 (11)	0.0133 (10)	0.0038 (9)	0.0029 (8)	0.0020 (8)
C8	0.0173 (11)	0.0202 (11)	0.0170 (11)	0.0007 (9)	0.0007 (8)	0.0011 (9)
C9	0.0211 (11)	0.0234 (12)	0.0144 (10)	0.0034 (9)	0.0011 (8)	−0.0024 (9)
C10	0.0225 (12)	0.0255 (12)	0.0150 (11)	0.0058 (10)	0.0046 (9)	0.0034 (9)
C11	0.0235 (12)	0.0219 (12)	0.0200 (11)	−0.0018 (10)	0.0064 (9)	0.0034 (9)
C12	0.0218 (11)	0.0211 (12)	0.0156 (11)	−0.0004 (9)	0.0029 (9)	−0.0014 (9)
C13	0.0384 (15)	0.0363 (15)	0.0172 (12)	−0.0014 (12)	0.0106 (11)	0.0014 (11)
C14	0.0176 (11)	0.0179 (11)	0.0162 (11)	−0.0008 (9)	0.0034 (8)	0.0001 (9)
C15	0.0165 (11)	0.0232 (12)	0.0175 (11)	0.0014 (9)	0.0041 (8)	0.0006 (9)
C16	0.0198 (11)	0.0230 (12)	0.0151 (10)	0.0000 (9)	0.0048 (8)	−0.0008 (9)
C17	0.0197 (11)	0.0172 (11)	0.0137 (10)	−0.0007 (9)	0.0044 (8)	−0.0009 (8)
C18	0.0173 (11)	0.0252 (12)	0.0120 (10)	0.0014 (9)	0.0021 (8)	−0.0011 (9)
C19	0.0192 (11)	0.0253 (12)	0.0177 (11)	−0.0014 (9)	0.0027 (9)	0.0022 (9)
C20	0.0241 (12)	0.0301 (13)	0.0195 (11)	0.0039 (10)	0.0020 (9)	0.0051 (10)
C21	0.0183 (11)	0.0389 (15)	0.0170 (11)	0.0026 (10)	0.0018 (9)	0.0002 (10)
C22	0.0192 (11)	0.0347 (14)	0.0186 (11)	−0.0056 (10)	0.0030 (9)	−0.0002 (10)
C23	0.0208 (11)	0.0237 (12)	0.0182 (11)	−0.0015 (10)	0.0033 (9)	0.0009 (9)
C24	0.0191 (13)	0.0513 (18)	0.0311 (14)	0.0039 (12)	−0.0009 (10)	0.0072 (13)
Cl1	0.272 (3)	0.0665 (9)	0.1016 (11)	0.0678 (12)	0.0716 (14)	−0.0032 (8)
C1A	0.038 (4)	0.125 (8)	0.080 (6)	0.025 (5)	−0.005 (4)	−0.074 (6)

### Geometric parameters (Å, °)

**Table:**

Zn1—N2	2.0326 (18)	C13—H13A	0.9800
Zn1—N2^i^	2.0327 (18)	C13—H13B	0.9800
Zn1—N1	2.0401 (18)	C13—H13C	0.9800
Zn1—N1^i^	2.0402 (18)	C14—C15	1.443 (3)
N1—C2	1.374 (3)	C15—C16	1.352 (3)
N1—C5	1.382 (3)	C15—H15A	0.9500
N2—C14	1.374 (3)	C16—C17	1.439 (3)
N2—C17	1.383 (3)	C16—H16A	0.9500
C1—C2	1.404 (3)	C17—C6^i^	1.401 (3)
C1—C14	1.406 (3)	C18—C23	1.396 (3)
C1—C18	1.494 (3)	C18—C19	1.395 (3)
C2—C3	1.443 (3)	C19—C20	1.389 (3)
C3—C4	1.352 (3)	C19—H19A	0.9500
C3—H3A	0.9500	C20—C21	1.392 (4)
C4—C5	1.440 (3)	C20—H20A	0.9500
C4—H4A	0.9500	C21—C22	1.386 (4)
C5—C6	1.400 (3)	C21—C24	1.509 (3)
C6—C17^i^	1.401 (3)	C22—C23	1.387 (3)
C6—C7	1.502 (3)	C22—H22A	0.9500
C7—C12	1.391 (3)	C23—H23A	0.9500
C7—C8	1.396 (3)	C24—H24A	0.9800
C8—C9	1.392 (3)	C24—H24B	0.9800
C8—H8A	0.9500	C24—H24C	0.9800
C9—C10	1.394 (3)	Cl1—C1A^ii^	1.507 (9)
C9—H9A	0.9500	Cl1—C1A	1.688 (8)
C10—C11	1.389 (3)	C1A—C1A^ii^	1.339 (14)
C10—C13	1.510 (3)	C1A—Cl1^ii^	1.507 (9)
C11—C12	1.396 (3)	C1A—H1AA	1.0000
C11—H11A	0.9500	C1A—H1AB	0.9592
C12—H12A	0.9500		
			
N2—Zn1—N2^i^	180.00 (9)	C10—C13—H13C	109.5
N2—Zn1—N1	90.04 (7)	H13A—C13—H13C	109.5
N2^i^—Zn1—N1	89.96 (7)	H13B—C13—H13C	109.5
N2—Zn1—N1^i^	89.96 (7)	N2—C14—C1	125.8 (2)
N2^i^—Zn1—N1^i^	90.04 (7)	N2—C14—C15	109.68 (19)
N1—Zn1—N1^i^	179.999 (1)	C1—C14—C15	124.5 (2)
C2—N1—C5	106.32 (18)	C16—C15—C14	107.1 (2)
C2—N1—Zn1	126.80 (14)	C16—C15—H15A	126.5
C5—N1—Zn1	126.87 (15)	C14—C15—H15A	126.5
C14—N2—C17	106.40 (18)	C15—C16—C17	107.5 (2)
C14—N2—Zn1	126.70 (14)	C15—C16—H16A	126.3
C17—N2—Zn1	126.74 (15)	C17—C16—H16A	126.3
C2—C1—C14	124.9 (2)	N2—C17—C6^i^	125.9 (2)
C2—C1—C18	117.52 (19)	N2—C17—C16	109.37 (19)
C14—C1—C18	117.63 (19)	C6^i^—C17—C16	124.7 (2)
N1—C2—C1	125.5 (2)	C23—C18—C19	118.0 (2)
N1—C2—C3	109.62 (19)	C23—C18—C1	121.5 (2)
C1—C2—C3	124.8 (2)	C19—C18—C1	120.6 (2)
C4—C3—C2	107.3 (2)	C20—C19—C18	120.7 (2)
C4—C3—H3A	126.4	C20—C19—H19A	119.6
C2—C3—H3A	126.4	C18—C19—H19A	119.6
C3—C4—C5	107.2 (2)	C19—C20—C21	121.2 (2)
C3—C4—H4A	126.4	C19—C20—H20A	119.4
C5—C4—H4A	126.4	C21—C20—H20A	119.4
N1—C5—C6	125.5 (2)	C22—C21—C20	117.9 (2)
N1—C5—C4	109.59 (19)	C22—C21—C24	120.9 (2)
C6—C5—C4	124.9 (2)	C20—C21—C24	121.2 (2)
C5—C6—C17^i^	124.9 (2)	C21—C22—C23	121.4 (2)
C5—C6—C7	117.89 (19)	C21—C22—H22A	119.3
C17^i^—C6—C7	117.16 (19)	C23—C22—H22A	119.3
C12—C7—C8	118.4 (2)	C22—C23—C18	120.8 (2)
C12—C7—C6	120.2 (2)	C22—C23—H23A	119.6
C8—C7—C6	121.4 (2)	C18—C23—H23A	119.6
C9—C8—C7	120.7 (2)	C21—C24—H24A	109.5
C9—C8—H8A	119.7	C21—C24—H24B	109.5
C7—C8—H8A	119.7	H24A—C24—H24B	109.5
C8—C9—C10	121.1 (2)	C21—C24—H24C	109.5
C8—C9—H9A	119.5	H24A—C24—H24C	109.5
C10—C9—H9A	119.5	H24B—C24—H24C	109.5
C11—C10—C9	118.0 (2)	C1A^ii^—Cl1—C1A	49.1 (5)
C11—C10—C13	120.9 (2)	C1A^ii^—C1A—Cl1^ii^	72.5 (8)
C9—C10—C13	121.1 (2)	C1A^ii^—C1A—Cl1	58.4 (6)
C10—C11—C12	121.3 (2)	Cl1^ii^—C1A—Cl1	130.9 (5)
C10—C11—H11A	119.4	C1A^ii^—C1A—H1AA	90.0
C12—C11—H11A	119.4	Cl1^ii^—C1A—H1AA	90.0
C7—C12—C11	120.6 (2)	Cl1—C1A—H1AA	90.0
C7—C12—H12A	119.7	C1A^ii^—C1A—H1AB	132.6
C11—C12—H12A	119.7	Cl1^ii^—C1A—H1AB	106.5
C10—C13—H13A	109.5	Cl1—C1A—H1AB	106.4
C10—C13—H13B	109.5	H1AA—C1A—H1AB	137.0
H13A—C13—H13B	109.5		
			
N2—Zn1—N1—C2	−0.55 (19)	C9—C10—C11—C12	−0.1 (4)
N2^i^—Zn1—N1—C2	179.45 (19)	C13—C10—C11—C12	−179.8 (2)
N2—Zn1—N1—C5	−178.92 (19)	C8—C7—C12—C11	−0.7 (3)
N2^i^—Zn1—N1—C5	1.08 (19)	C6—C7—C12—C11	178.6 (2)
N1—Zn1—N2—C14	−4.16 (19)	C10—C11—C12—C7	0.5 (4)
N1^i^—Zn1—N2—C14	175.84 (19)	C17—N2—C14—C1	−178.3 (2)
N1—Zn1—N2—C17	−178.82 (19)	Zn1—N2—C14—C1	6.2 (3)
N1^i^—Zn1—N2—C17	1.18 (19)	C17—N2—C14—C15	1.3 (3)
C5—N1—C2—C1	−177.5 (2)	Zn1—N2—C14—C15	−174.23 (15)
Zn1—N1—C2—C1	3.9 (3)	C2—C1—C14—N2	−2.4 (4)
C5—N1—C2—C3	1.6 (3)	C18—C1—C14—N2	177.3 (2)
Zn1—N1—C2—C3	−177.08 (15)	C2—C1—C14—C15	178.1 (2)
C14—C1—C2—N1	−3.0 (4)	C18—C1—C14—C15	−2.2 (3)
C18—C1—C2—N1	177.3 (2)	N2—C14—C15—C16	−1.8 (3)
C14—C1—C2—C3	178.1 (2)	C1—C14—C15—C16	177.8 (2)
C18—C1—C2—C3	−1.5 (3)	C14—C15—C16—C17	1.4 (3)
N1—C2—C3—C4	−2.3 (3)	C14—N2—C17—C6^i^	−178.2 (2)
C1—C2—C3—C4	176.7 (2)	Zn1—N2—C17—C6^i^	−2.6 (3)
C2—C3—C4—C5	2.0 (3)	C14—N2—C17—C16	−0.4 (3)
C2—N1—C5—C6	179.0 (2)	Zn1—N2—C17—C16	175.12 (15)
Zn1—N1—C5—C6	−2.4 (3)	C15—C16—C17—N2	−0.7 (3)
C2—N1—C5—C4	−0.3 (3)	C15—C16—C17—C6^i^	177.1 (2)
Zn1—N1—C5—C4	178.34 (15)	C2—C1—C18—C23	118.4 (2)
C3—C4—C5—N1	−1.1 (3)	C14—C1—C18—C23	−61.2 (3)
C3—C4—C5—C6	179.6 (2)	C2—C1—C18—C19	−61.1 (3)
N1—C5—C6—C17^i^	3.6 (4)	C14—C1—C18—C19	119.2 (2)
C4—C5—C6—C17^i^	−177.3 (2)	C23—C18—C19—C20	−1.9 (3)
N1—C5—C6—C7	−176.6 (2)	C1—C18—C19—C20	177.7 (2)
C4—C5—C6—C7	2.5 (3)	C18—C19—C20—C21	1.3 (4)
C5—C6—C7—C12	113.2 (2)	C19—C20—C21—C22	0.3 (4)
C17^i^—C6—C7—C12	−67.0 (3)	C19—C20—C21—C24	−179.7 (2)
C5—C6—C7—C8	−67.5 (3)	C20—C21—C22—C23	−1.3 (4)
C17^i^—C6—C7—C8	112.3 (2)	C24—C21—C22—C23	178.7 (2)
C12—C7—C8—C9	0.6 (3)	C21—C22—C23—C18	0.7 (4)
C6—C7—C8—C9	−178.7 (2)	C19—C18—C23—C22	0.9 (3)
C7—C8—C9—C10	−0.2 (3)	C1—C18—C23—C22	−178.7 (2)
C8—C9—C10—C11	−0.1 (3)	C1A^ii^—Cl1—C1A—Cl1^ii^	0.0
C8—C9—C10—C13	179.7 (2)		

Symmetry codes: (i) −*x*+1, −*y*+1, −*z*+1; (ii) −*x*, −*y*+2, −*z*+1.
